# Supplementary material for: Psychosocial Determinants of COVID-19 Vaccine Hesitancy and the Mediating Role of Various Attitudes towards Science
Source: Vaccines (Basel). 2023 Jul 31;11(8):1310. doi: 10.3390/vaccines11081310 (PMC10459256; doi:10.3390/vaccines11081310)
Supplement: Supplementary file 1 [file vaccines-11-01310-s001.zip › vaccines-2459249-supplementary.pdf]

S1. Study Materials

Survey Flow

Block: Electronic Consent
Standard: Prolific ID

BlockRandomizer: 3 - Evenly Present Elements

Standard: Reactance Proneness
Standard: Ostracism
Block: DOG Scale

BlockRandomizer: 3 - Evenly Present Elements

Standard: Vaccine Info
Standard: Attitudes Toward Science
Standard: Trust Questionnaire

Standard: Sociodemographic Variables
Standard: Prolific redirect

Page Break

Start of Block: Prolific ID

prolific_id Before we begin, please enter your Prolific ID here. This will help us keep track of your participation.

____

End of Block: Prolific ID

Reactance Proneness

rp_time Timing
First Click (1)
Last Click (2)
Page Submit (3)
Click Count (4)

---

rp_ins **Instructions**
Indicate your agreement with the following statements.

---

rp1 Regulations trigger a sense of resistance in me.

- Strongly disagree (1)
- Somewhat disagree (2)
- Neither agree nor disagree (3)
- Somewhat agree (4)
- Strongly agree (5)

---

rp2 I find contradicting others stimulating.

- Strongly disagree (1)
- Somewhat disagree (2)
- Neither agree nor disagree (3)
- Somewhat agree (4)
- Strongly agree (5)

---

rp3 When something is prohibited, I usually think "That's exactly what I am going to do."

- Strongly disagree (1)
- Somewhat disagree (2)
- Neither agree nor disagree (3)
- Somewhat agree (4)
- Strongly agree (5)

---

rp4 I consider advice from others to be an intrusion.

- Strongly disagree (1)
- Somewhat disagree (2)
- Neither agree nor disagree (3)
- Somewhat agree (4)
- Strongly agree (5)

---

rp5 I become frustrated when I am unable to make free and independent decisions.

- Strongly disagree (1)
- Somewhat disagree (2)
- Neither agree nor disagree (3)
- Somewhat agree (4)
- Strongly agree (5)

rp6 It irritates me when someone points out things which are obvious to me.

- Strongly disagree (1)
- Somewhat disagree (2)
- Neither agree nor disagree (3)
- Somewhat agree (4)
- Strongly agree (5)

rp7 I become angry when my freedom of choice is restricted.

- Strongly disagree (1)
- Somewhat disagree (2)
- Neither agree nor disagree (3)
- Somewhat agree (4)
- Strongly agree (5)

rp8 Advice and recommendations induce me to do just the opposite.

- Strongly disagree (1)
- Somewhat disagree (2)
- Neither agree nor disagree (3)
- Somewhat agree (4)
- Strongly agree (5)

---

rp9 I resist the attempts of others to influence me.

- Strongly disagree (1)
- Somewhat disagree (2)
- Neither agree nor disagree (3)
- Somewhat agree (4)
- Strongly agree (5)

---

rp10 It makes me angry when another person is held up as a model for me to follow.

- Strongly disagree (1)
- Somewhat disagree (2)
- Neither agree nor disagree (3)
- Somewhat agree (4)
- Strongly agree (5)

rp11 When someone forces me to do something, I feel like doing the opposite.

- ○ Strongly disagree (1)
- ○ Somewhat disagree (2)
- ○ Neither agree nor disagree (3)
- ○ Somewhat agree (4)
- ○ Strongly agree (5)

End of Block: Reactance Proneness

Start of Block: Ostracism

Ostracism

ost_time Timing
First Click (1)
Last Click (2)
Page Submit (3)
Click Count (4)

---

ost_ins **Instructions**
The following questions will ask about your sense of how you and your community are perceived. There are no right or wrong answers, we are interested in understanding your own experience.

---

ost1 My community is excluded from many of the important decisions being made today.

- ○ Strongly disagree (1)
- ○ Disagree (2)
- ○ Somewhat disagree (3)
- ○ Neither agree nor disagree (4)
- ○ Somewhat agree (5)
- ○ Agree (6)
- ○ Strongly agree (7)

---

ost2 My community and I are often the last to receive help.

- Strongly disagree (1)
- Disagree (2)
- Somewhat disagree (3)
- Neither agree nor disagree (4)
- Somewhat agree (5)
- Agree (6)
- Strongly agree (7)

---

ost3 I feel that the values and things I hold dear are threatened by the way the world is changing.

- Strongly disagree (1)
- Disagree (2)
- Somewhat disagree (3)
- Neither agree nor disagree (4)
- Somewhat agree (5)
- Agree (6)
- Strongly agree (7)

---

ost4 The concerns of my community are ignored by those in power.

- Strongly disagree (1)
- Disagree (2)
- Somewhat disagree (3)
- Neither agree nor disagree (4)
- Somewhat agree (5)
- Agree (6)
- Strongly agree (7)

---

ost5 The insights that my community and I have are often ignored.

- Strongly disagree (1)
- Disagree (2)
- Somewhat disagree (3)
- Neither agree nor disagree (4)
- Somewhat agree (5)
- Agree (6)
- Strongly agree (7)

---

ost6 My community and I are generally held in high esteem.

- Strongly disagree (7)
- Disagree (6)
- Somewhat disagree (5)
- Neither agree nor disagree (4)
- Somewhat agree (3)
- Agree (2)
- Strongly agree (1)

---

ost7 I feel that my community is often mocked.

- Strongly disagree (1)
- Disagree (2)
- Somewhat disagree (3)
- Neither agree nor disagree (4)
- Somewhat agree (5)
- Agree (6)
- Strongly agree (7)

---

ost8 I feel that my community and those I hold dear are being left behind as society changes.

- Strongly disagree (1)
- Disagree (2)
- Somewhat disagree (3)
- Neither agree nor disagree (4)
- Somewhat agree (5)
- Agree (6)
- Strongly agree (7)

End of Block: Ostracism

Start of Block: DOG Scale

Dogmatism

dog_ins
We are interested in your personal opinion on each of the following statements.

When you read each one, please use the scale to tell us whether, in general, you disagree or agree.

---

dogx I may be wrong about some of the little things in life, but I am quite certain I am right about all the BIG issues.

	Strongly Disagree				Neutral				Strongly agree
	-4	-3	-2	-1	0	1	2	3	4
1 ()									

---

dogy Someday I will probably think that many of my present ideas were wrong.

	Strongly Disagree				Neutral			Strongly agree	
	-4	-3	-2	-1	0	1	2	3	4
1 ()									

---

dog1 Anyone who is honestly and truly seeking the truth will end up believing what I believe.

	Strongly Disagree				Neutral			Strongly agree	
	-4	-3	-2	-1	0	1	2	3	4
1 ()									

---

dog2 There are so many things we have not discovered yet, nobody should be absolutely certain his/her beliefs are right.

	Strongly Disagree				Neutral			Strongly agree	
	-4	-3	-2	-1	0	1	2	3	4
1 ()									

---

dog3 The things I believe in are so completely true, I could never doubt them.

	Strongly Disagree				Neutral			Strongly agree	
	-4	-3	-2	-1	0	1	2	3	4
1 ()									

dog4 I have never discovered a system of beliefs that explains everything to my satisfaction.

	Strongly Disagree				Neutral				Strongly agree
	-4	-3	-2	-1	0	1	2	3	4
1 ()									

dog5 It is best to be open to all possibilities and ready to reevaluate all your beliefs.

	Strongly Disagree				Neutral				Strongly agree
	-4	-3	-2	-1	0	1	2	3	4
1 ()									

dog6 My opinions are right and will stand the test of time.

	Strongly Disagree				Neutral				Strongly agree
	-4	-3	-2	-1	0	1	2	3	4
1 ()									

dog7 Flexibility is a real virtue in thinking, since you may well be wrong.

	Strongly Disagree				Neutral				Strongly agree
	-4	-3	-2	-1	0	1	2	3	4

1 ()	

dog8 My opinions and beliefs fit together perfectly to make a crystal-clear “picture” of things.

	Strongly Disagree				Neutral				Strongly agree
	-4	-3	-2	-1	0	1	2	3	4
1 ()									

dog9 There are no discoveries or facts that could possibly make me change my mind about the things that matter most in life.

	Strongly Disagree				Neutral				Strongly agree
	-4	-3	-2	-1	0	1	2	3	4
1 ()									

dog10 I am a long way from reaching final conclusions about the central issues in life.

	Strongly Disagree				Neutral				Strongly agree
	-4	-3	-2	-1	0	1	2	3	4
1 ()									

dog11 The person who is absolutely certain she/he has the truth will probably never find it.

	Strongly Disagree				Neutral			Strongly agree	
	-4	-3	-2	-1	0	1	2	3	4
1 ()									

dog12 I am absolutely certain that my ideas about the fundamental issues in life are correct.

	Strongly Disagree				Neutral			Strongly agree	
	-4	-3	-2	-1	0	1	2	3	4
1 ()									

dog13 The people who disagree with me may well turn out to be right.

	Strongly Disagree				Neutral			Strongly agree	
	-4	-3	-2	-1	0	1	2	3	4
1 ()									

dog14 I am so sure I am right about the important things in life, there is no evidence that could convince me otherwise.

	Strongly Disagree				Neutral			Strongly agree	
	-4	-3	-2	-1	0	1	2	3	4
1 ()									

dog15 If you are “open-minded” about the most important things in life, you will probably reach the wrong conclusions.

	Strongly Disagree				Neutral				Strongly agree
	-4	-3	-2	-1	0	1	2	3	4
1 ()									

dog16 Twenty years from now, some of my opinions about the important things in life will probably have changed.

	Strongly Disagree				Neutral				Strongly agree
	-4	-3	-2	-1	0	1	2	3	4
1 ()									

dog17 “Flexibility in thinking” is another name for being “wishy-washy.”

	Strongly Disagree				Neutral				Strongly agree
	-4	-3	-2	-1	0	1	2	3	4
1 ()									

dog18 No one knows all the essential truths about the central issues in life.

	Strongly Disagree				Neutral				Strongly agree
	-4	-3	-2	-1	0	1	2	3	4

1 ()	

dog19 Someday I will probably realize my present ideas about the BIG issues are wrong.

	Strongly Disagree				Neutral			Strongly agree	
	-4	-3	-2	-1	0	1	2	3	4
1 ()									

dog20 People who disagree with me are just plain wrong and often evil as well.

	Strongly Disagree				Neutral			Strongly agree	
	-4	-3	-2	-1	0	1	2	3	4
1 ()									

End of Block: DOG Scale

Vaccine Info

vax_time Timing
First Click (1)
Last Click (2)
Page Submit (3)
Click Count (4)

---

vax_ins *We recognize that people are responding to COVID-19 in a variety of different ways. Our aim is to understand your experiences as you are navigating this difficult time. From our perspective, there are no right or wrong answers here. Please answer the following questions and remember that all of your responses are anonymous.*

---

vax1 As of today, have you been vaccinated for COVID-19?

- ○ No (1)
- ○ Yes (2)

Display This Question:

If vax1 = 2

vax_y1 From the time you were eligible, how long did it take you to get vaccinated?

- ○ I signed up immediately (1)
- ○ a few days (2)
- ○ a few weeks (3)
- ○ a few months (4)
- ○ many months (5)

Display This Question:

If vax1 = 2

vax_y2 What was the most important factor you were considering when you decided whether or not to be vaccinated? (Please write)

____

Display This Question:

If vax1 = 2

vax_y3 How hesitant did you feel about getting vaccinated?

- ○ 1 (Not at all) (1)
- ○ 2 (2)
- ○ 3 (3)
- ○ 4 (4)
- ○ 5 (5)
- ○ 6 (6)
- ○ 7 (Very hesitant) (7)

Display This Question:

If vax1 = 2

vax_y4 What were the most important reasons for your level of comfort with getting the COVID-19 vaccine? (If you have multiple reasons, please write them in order of importance to you)

____

Display This Question:

If vax1 = 1

vax_n1 How hesitant do you feel about getting vaccinated?

- 1 (Not at all) (1)
- 2 (2)
- 3 (3)
- 4 (4)
- 5 (5)
- 6 (6)
- 7 (Very hesitant) (7)

Display This Question:

If vax1 = 1

vax_n2 How long have you been eligible to get the vaccine?

- Still not eligible (1)
- A few days (2)
- A few weeks (3)
- A few months (4)

Display This Question:

If vax1 = 1

vax_n3 Do you intend to get the vaccine at some point?

- 1 (Never) (1)
- 2 (2)
- 3 (3)
- 4 (4)
- 5 (5)
- 6 (6)
- 7 (Probably eventually) (7)

Display This Question:

If vax1 = 1

vax_n4 What are the most important factors you consider when deciding whether or not to be vaccinated? (If you have multiple reasons, please write them in order of importance to you)

____

Attitudes Toward Science

sciatt_time Timing
First Click (1)
Last Click (2)
Page Submit (3)
Click Count (4)

---

sciatt_ins **Instructions**
The following questions are about your personal attitudes or perspectives towards science. There are no right or wrong answers and all of your responses are anonymous.

---

sciatt1 Science and technology make life healthier, easier, and more comfortable.

- Strongly disagree (1)
- Somewhat disagree (2)
- Neither agree nor disagree (3)
- Somewhat agree (4)
- Strongly agree (5)

---

sciatt2 We depend too much on scientific expertise and not enough on personal experience.

- Strongly disagree (5)
- Somewhat disagree (4)
- Neither agree nor disagree (3)
- Somewhat agree (2)
- Strongly agree (1)

---

sciatt3 Science makes life change too fast.

- Strongly disagree (5)
- Somewhat disagree (4)
- Neither agree nor disagree (3)
- Somewhat agree (2)
- Strongly agree (1)

---

sciatt4 It is not important for me to know about science in my daily life.

- Strongly disagree (5)
- Somewhat disagree (4)
- Neither agree nor disagree (3)
- Somewhat agree (2)
- Strongly agree (1)

---

sciatt5 Science and technology will create more opportunities for the next generation.

- Strongly disagree (1)
- Somewhat disagree (2)
- Neither agree nor disagree (3)
- Somewhat agree (4)
- Strongly agree (5)

sciatt6 On balance, the benefits of scientific research have outweighed the harmful results.

- Strongly disagree (1)
- Somewhat disagree (2)
- Neither agree nor disagree (3)
- Somewhat agree (4)
- Strongly agree (5)

sciatt7 Science is too dogmatic.

- Strongly disagree (5)
- Somewhat disagree (4)
- Neither agree nor disagree (3)
- Somewhat agree (2)
- Strongly agree (1)

sciatt8 Science does not pay enough attention to the wisdom of nonscientists.

- Strongly disagree (5)
- Somewhat disagree (4)
- Neither agree nor disagree (3)
- Somewhat agree (2)
- Strongly agree (1)

---

sciatt9 Science is too biased by the values or personal interests of researchers

- Strongly disagree (5)
- Somewhat disagree (4)
- Neither agree nor disagree (3)
- Somewhat agree (2)
- Strongly agree (1)

---

sciatt10 Science is too biased by the financial interests of researchers

- ○ Strongly disagree (5)
- ○ Somewhat disagree (4)
- ○ Neither agree nor disagree (3)
- ○ Somewhat agree (2)
- ○ Strongly agree (1)

End of Block: Attitudes Toward Science

Trust Questionnaire

tr_time Timing
First Click (1)
Last Click (2)
Page Submit (3)
Click Count (4)

---

trust_ins **Instructions**
Please rate your feelings of trust towards the following people and organizations using the scale below.

---

tr_sci How much do you trust the following groups of scientists:

	**Do not trust at all**	**Trust completely**
	0 1 2 3 4 5 6 7 8 9 10	
Science in general ()		
Physicists ()		
Chemists ()		
Biologists ()		
Psychologists ()		
Social Scientists ()		

---

tr_med How much do you trust the following medical scientists:

	**Do not trust at all**	**Trust completely**
	0 1 2 3 4 5 6 7 8 9 10	

Medical science in general ()	
Your Family Doctor ()	
Public Health Researchers ()	
Epidemiologists ()	
Public Health Institutions (e.g., NIH or the CDC) ()	
Pharmaceutical Researchers ()	

End of Block: Trust Questionnaire

Sociodemographic Variables

sdv_time Timing
First Click (1)
Last Click (2)
Page Submit (3)
Click Count (4)

---

sdv_ins **Instructions**
This final page contains some basic demographic questions. Please select the response that feels most appropriate for you.

Remember that all of your responses are anonymous and confidential.

---

gender What is your gender?

- ○ Male (1)
- ○ Female (2)
- ○ Other (3)
- ○ Rather not say (4)

---

*

age How old are you (in years)?

____

---

edu What is the highest level of education that you have completed?

- ○ Grade school (1)
- ○ Some high school (2)
- ○ High school grad (3)
- ○ Some college (4)
- ○ College grad (5)
- ○ Masters degree (6)
- ○ PhD (7)

---

eth How would you describe your ethnicity or race? [check all that apply]

- ▢ Black/African (1)
- ▢ East Asian (2)
- ▢ Hispanic (3)
- ▢ Middle Eastern (4)
- ▢ Native American (5)
- ▢ South Asian (6)
- ▢ White/European (7)
- ▢ Other (8)
- ▢ Would rather not say (9)

rel_aff What is your primary religious or spiritual affiliation?

- Agnostic (1)
- Atheist (2)
- Bahá'í (3)
- Buddhist (4)
- Christian (5)
- Confucian (6)
- Daoist (7)
- Hindu (13)
- Humanist (8)
- Jewish (9)
- Muslim (10)
- New Age (11)
- None (14)
- Sikh (12)
- Other. Please Describe. (15) ____

rel_imp How important is your religiosity to you?

- Not at all important (1)
- Slightly important (2)
- Moderately important (3)
- Very important (4)
- Extremely important (5)

---

pol_aff What is your primary political affiliation?

- Democrat (1)
- Republican (2)
- Libertarian (3)
- Independent (7)
- Green Party (4)
- No Political Party or Affiliation (5)
- Other Political Party or Affiliation. Please Write. (6) ____

---

pol_spec When it comes to politics in the US, where do you typically place yourself on the political spectrum?

- Very Liberal (1)
- Liberal (2)
- Slightly Liberal (3)
- Moderate (4)
- Slightly Conservative (5)
- Conservative (6)
- Very Conservative (7)

---

ses This ladder represents where people stand in society. At the top of the ladder (10) are the people who are the best off, those who have the most money, most education, and best jobs. At the bottom (1) are the people who are the worst off, those who have the least money, least education, worst jobs, or no job.

Please select the rung that best represents where you think you stand on the ladder.

- ○ 10 (Best) (10)
- ○ 9 (9)
- ○ 8 (8)
- ○ 7 (7)
- ○ 6 (6)
- ○ 5 (5)
- ○ 4 (4)
- ○ 3 (3)
- ○ 2 (2)
- ○ 1 (Worst) (1)

---

*

state What state do you live in?

____

End of Block: Sociodemographic Variables

Prolific redirect

Prolific_redirect Thank you for taking the time to complete our study.

Please follow this link back to Prolific in order to confirm that you completed the study: https://app.prolific.co/submissions/complete?cc=XXXXXXXXX

Before you connect back to Prolific, please feel free to share any thoughts you might have about this study below.

We hope that this was a positive experience for you!

____

End of Block: Prolific redirect

S2. Exploratory Factor Analyses

EFA: Attitudes towards and trust in science

Kaiser-Meyer-Olkin factor adequacy
Call: KMO(r = attwdsci_cor)
Overall MSA = 0.96
MSA for each item =

atsci1	atsci2	atsci3	atsci4	atsci5
0.95	0.96	0.96	0.96	0.96
atsci6	atsci7	atsci8	atsci9	atsci10
0.97	0.97	0.96	0.95	0.94
sci_gen	physicists	chemists	biologists	psychologists
0.97	0.96	0.94	0.96	0.93
social_scientists	med_gen	family_doc	pub_health	epidemiologists
0.93	0.97	0.97	0.95	0.96
NIH_CDC	Pharma			
0.97	0.97			

R was not square, finding R from data
$chisq
[1] 18030.74
$p.value
[1] 0

$df
[1] 231

[1] 3.003413e-09

Figure S1. Scree Plots for Attitudes towards and Trust in Science EFA

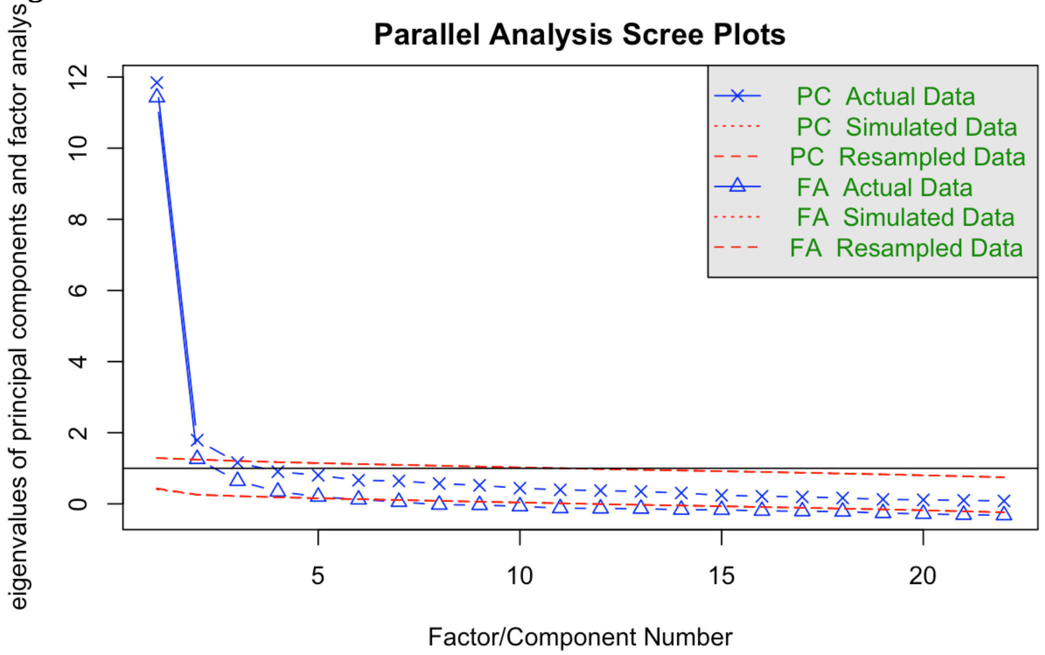

Parallel analysis suggests that the number of factors = 5 and the number of components = 2.

Factor Analysis using method = pa
Call: fa(r = attwdsci_data, nfactors = 5, rotate = "oblimin", SMC = TRUE,

```
    covar = FALSE, max.iter = 100, fm = "pa")
Standardized loadings (pattern matrix) based upon correlation matrix

                       PA1  PA3  PA2  PA4  PA5
SS loadings           3.87 3.55 3.22 2.15 2.01
Proportion Var        0.18 0.16 0.15 0.10 0.09
Cumulative Var        0.18 0.34 0.48 0.58 0.67
Proportion Explained  0.26 0.24 0.22 0.15 0.14
Cumulative Proportion 0.26 0.50 0.72 0.86 1.00

 With factor correlations of
     PA1  PA3  PA2  PA4  PA5
PA1 1.00 0.69 0.47 0.58 0.62
PA3 0.69 1.00 0.57 0.53 0.72
PA2 0.47 0.57 1.00 0.56 0.45
PA4 0.58 0.53 0.56 1.00 0.40
PA5 0.62 0.72 0.45 0.40 1.00

Mean item complexity =  1.4
Test of the hypothesis that 5 factors are sufficient.

The degrees of freedom for the null model are  231  and the objective function was  19.62 with Chi Square of  18030.74
The degrees of freedom for the model are 131  and the objective function was  0.8

The root mean square of the residuals (RMSR) is  0.02
The df corrected root mean square of the residuals is  0.03

The harmonic number of observations is  910 with the empirical chi square  149.03  with prob <  0.13
The total number of observations was  928  with Likelihood Chi Square =  733.14  with prob <  1.8e-84

Tucker Lewis Index of factoring reliability =  0.94
RMSEA index =  0.07  and the 90 % confidence intervals are  0.065 0.075
BIC =  -161.99
Fit based upon off diagonal values = 1
Measures of factor score adequacy
                                                   PA1  PA3  PA2  PA4  PA5
Correlation of (regression) scores with factors   0.98 0.96 0.94 0.90 0.95
Multiple R square of scores with factors          0.96 0.93 0.89 0.81 0.90
Minimum correlation of possible factor scores     0.91 0.86 0.78 0.63 0.80
```

Table S1. Factor Loadings for EFA with all Attitudes towards and Trust in Science items.

	PA1	PA3	PA2	PA4	PA5
atsci1	-.01	.04	-.02	.78	.06
atsci2	.15	.05	.58	.20	-.11
atsci3	.18	-.16	.27	.43	-.04
atsci4	.20	-.09	.12	.23	0.01
atsci5	-.10	.08	.03	.65	.10
atsci6	.09	.04	.21	.47	-.11
atsci7	.14	-.06	.56	.16	.01
atsci8	.18	-.07	.74	.03	.01
atsci9	-.07	.16	.72	.00	.14
atsci10	-.10	.20	.72	-.06	.10
tr_sci_gen	.63	.16	.06	.07	.12

tr_physicists	.85	.06	.03	.02	.01
tr_chemists	.89	.02	.04	-.03	.07
tr_biologists	.72	.10	.03	.06	.15
tr_psychologists	.11	.00	-.06	.07	.82
tr_soc_scientists	.02	.02	.10	.00	.83
tr_med_gen	.37	.50	.02	.13	-.02
tr_fam_doc	.20	.47	-.06	.11	.03
tr_pub_health	.12	.75	.03	.04	.06
tr_epidemiologists	.27	.63	.02	.11	.00
tr_NIH_CDC	-.05	.70	.13	.07	.12
tr_pharma	-.03	.70	.09	-.01	.07

EFA: Perceived Communal Ostracism

Kaiser-Meyer-Olkin factor adequacy
Call: KMO(r = ost_cor)
Overall MSA = 0.88
MSA for each item =
ost1 ost2 ost3 ost4 ost5 ost6 ost7 ost8
0.91 0.90 0.84 0.84 0.84 0.93 0.92 0.88
R was not square, finding R from data
$chisq
[1] 4174.471

$p.value
[1] 0

$df
[1] 28

[1] 0.01088607

Figure S2. Scree Plots for Perceived Communal Ostracism

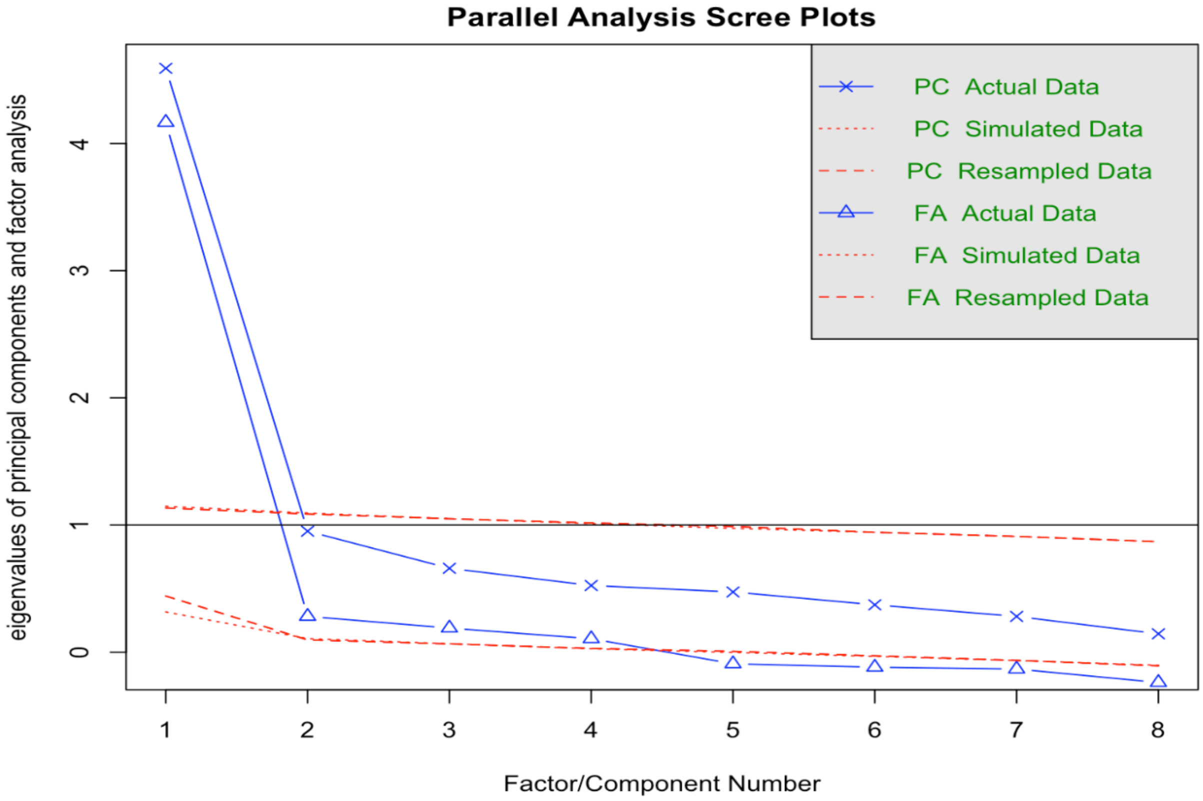

Parallel analysis suggests that the number of factors = 2 and the number of components = 1

Factor Analysis using method = pa

```
Call: fa(r = ost_data, nfactors = 2, rotate = "varimax", SMC = TRUE,
   covar = FALSE, max.iter = 100, fm = "pa")
Standardized loadings (pattern matrix) based upon correlation matrix

                    PA1  PA2
SS loadings           3.42 1.38
Proportion Var        0.43 0.17
Cumulative Var        0.43 0.60
Proportion Explained  0.71 0.29
Cumulative Proportion 0.71 1.00

Mean item complexity =  1.3
Test of the hypothesis that 2 factors are sufficient.

The degrees of freedom for the null model are  28  and the objective function was  4.52 with Chi Square of  4174.47
The degrees of freedom for the model are 13  and the objective function was  0.38

The root mean square of the residuals (RMSR) is  0.04
The df corrected root mean square of the residuals is  0.06

The harmonic number of observations is  922 with the empirical chi square  86.29  with prob <  7.1e-13
The total number of observations was  928  with Likelihood Chi Square =  348.99  with prob <  1.3e-66

Tucker Lewis Index of factoring reliability =  0.825
RMSEA index =  0.167  and the 90 % confidence intervals are  0.152 0.182
BIC =  260.16
Fit based upon off diagonal values = 0.99
Measures of factor score adequacy
                                                  PA1  PA2
Correlation of (regression) scores with factors   0.93 0.85
Multiple R square of scores with factors          0.86 0.72
Minimum correlation of possible factor scores     0.72 0.44
```

Table S2. Factor Loadings for perceived Communal Ostracism EFA.

	PA1	PA2
ost1	.75	.29
ost2	.76	.19
ost3	.16	.83
ost4	.76	.33
ost5	.81	.32
ost6	.57	.10
ost7	.66	.23
ost8	.50	.54

S3. Analyses with Social Science Trust included

Table S3. Saturated Path Analysis including Trust in Social Science

Variables	*B*	SE	z-score	β	p

**Outcome: COVID-19 Vaccine Hesitancy (without mediators)**					
Education	-.11	.07	-1.50	-.05	.134
SES	-.20	.05	-4.38	-.14	<.001
Religiosity	.16	.06	2.87	.09	.004
RP	.62	.11	5.76	.18	<.001
Dogmatism	.09	.06	1.40	.04	.161
Com Ost	.05	.06	.91	.03	.366
Political Orientation	.63	.05	13.99	.46	<.001
**Outcome: Epistemic Confidence**					
Education	.04	.03	1.27	.04	.203
SES	-.00	.02	-.02	-.00	.986
Religiosity	-.12	.02	-5.33	-.17	< .001
RP	-.41	.05	-8.97	-.27	< .001
Dogmatism	-.07	.03	-2.75	-.08	.006
Com Ost	-.09	.02	-3.83	-.12	< .001
Political Orientation	-.22	.02	-11.41	-.37	< .001
**Outcome: Belief that Science and Technology are Beneficial**					
Education	.07	.02	2.89	.10	.004
SES	.01	.02	.93	.03	.352
Religiosity	-.04	.02	-2.32	-.08	.020
RP	-.23	.04	-6.39	-.21	< .001
Dogmatism	-.11	.02	-5.45	-.18	< .001
Com Ost	-.03	.02	-1.61	-.05	.106
Political Orientation	-.11	.02	-7.57	-.27	< .001
**Outcome: Trust in Science in General**					
Education	.25	.07	3.55	.12	< .001
SES	.06	.05	1.35	.05	.176
Religiosity	-.21	.05	-3.81	-.13	< .001
RP	-.55	.11	-5.17	-.17	< .001
Dogmatism	-.25	.06	-4.16	-.14	< .001
Com Ost	-.15	.06	-2.68	-.09	.007
Political Orientation	-.33	.04	-7.48	-.27	< .001
**Outcome: Trust in Medical Science**					
Education	.12	.07	1.63	.05	.104
SES	.13	.05	2.92	.10	.003
Religiosity	-.07	.06	-1.31	.04	.191
RP	-.72	.11	-6.73	-.21	< .001
Dogmatism	-.21	.06	-3.55	-.11	< .001
Com Ost	-.22	.06	-4.03	-.13	< .001
Political Orientation	-.51	.05	-11.47	-.39	< .001
**Outcome: Trust in Social Science**					
Education	-.06	.08	-.76	-.03	.445
SES	.15	.05	2.75	.09	.006
Religiosity	-.01	.06	-.16	-.01	.871
RP	-.75	.12	-6.05	-.20	<.001

Dogmatism	-.16	.07	-2.22	-.07	.026
Com Ost	-.23	.06	-3.53	-.12	<.001
Political Orientation	-.61	.05	-11.81	-.41	<.001
**Outcome: COVID-19 Vaccine Hesitancy**					
Education	-.02	.06	-.36	-.01	.716
SES	-.16	.04	-4.00	-.11	<.001
Religiosity	.08	.05	1.50	.04	.133
RP	.19	.10	1.95	.06	.051
Dogmatism	-.04	.05	-.70	-.02	.485
Com Ost	-.06	.05	-1.20	-.03	.232
Political Orientation	.38	.05	8.49	.28	<.001
Epistemic Confidence	-.42	.10	-4.23	-.18	<.001
Sci & Tech Beneficial	-.25	.12	-2.12	-.08	.034
Trust in General Sci	.03	.05	.66	.03	.507
Trust in Medical Sci	-.44	.05	-8.18	-.43	<.001
Trust in Social Sci	.13	.04	3.46	.14	.001
